# Supplementary material for: Chemoproteomics validates selective targeting of Plasmodium M1 alanyl aminopeptidase as an antimalarial strategy
Source: eLife. 2024 Jul 8;13:RP92990. doi: 10.7554/eLife.92990 (PMC11230628; doi:10.7554/eLife.92990)
Supplement: Figure 1—source data 1. [file elife-92990-fig1-data1.docx]

**Figure 1-source data 1.** Data collection and refinement statistics for X-ray crystal structure.

|  | **M1 MIPS2673** |
| --- | --- |
| PDB ID | 8SLO |
| *Data collection statistics* | |
| Wavelength | 0.953700 |
| Resolution range | 41.4 - 1.55 (1.605 - 1.55) |
| Space group | P 21 21 21 |
| Unit cell | 76.4 108.5 117.6 90 90 90 |
| Total reflections | 1038039 (39097) |
| Unique reflections | 139690 (12944) |
| Multiplicity | 7.4 (6.4) |
| Completeness (%) | 98.39 (91.92) |
| Mean I/sigma(I) | 8.5 (0.9) |
| Wilson B-factor | 17.19 |
| R-pim (all I+ & I-) | 0.057 (0.772) |
| CC1/2 | 0.997 (0.385) |
| *Data Refinement statistics* | |
| Reflections used in refinement | 139628 (12905) |
| Reflections used for R-free | 7039 (646) |
| R-work | 0.1712 (0.2886) |
| R-free | 0.2021 (0.3072) |
| Number of non-hydrogen atoms | 8774 |
| macromolecules | 7432 |
| ligands | 45 |
| solvent | 1297 |
| Protein residues | 889 |
| RMS(bonds) | 0.007 |
| RMS(angles) | 0.88 |
| Ramachandran favored (%) | 98.20 |
| Ramachandran allowed (%) | 1.69 |
| Ramachandran outliers (%) | 0.11 |
| Rotamer outliers (%) | 0.24 |
| Clashscore | 2.35 |
| Average B-factor | 21.92 |
| macromolecules | 20.04 |
| ligands | 19.42 |
| solvent | 32.76 |

Statistics for the highest-resolution shell are shown in parentheses.
